# Waste-to-Carbon: Is the Torrefied Sewage Sludge with High Ash Content a Better Fuel or Fertilizer?

**DOI:** 10.3390/ma13040954

**Published:** 2020-02-20

**Authors:** Jakub Pulka, Piotr Manczarski, Paweł Stępień, Marzena Styczyńska, Jacek A. Koziel, Andrzej Białowiec

**Affiliations:** 1 Faculty of Agronomy and Bioengineering, Poznan University of Life Sciences, 28 Wojska Polskiego Str., 60-637 Poznań, Poland; jakub.pulka@up.poznan.pl; 2 Department of Environmental Engineering, Faculty of Building Services, Hydro and Environmental Engineering, Warsaw University of Technology, 20 Nowowiejska Str., 00-653 Warszawa, Poland; piotr.manczarski@pw.edu.pl; 3 Institute of Agricultural Engineering, Faculty of Life Sciences and Technology, Wroclaw University of Environmental and Life Sciences, 37a Chełmońskiego Str., 51-630 Wroclaw, Poland; pawel.stepien@upwr.edu.pl; 4 Faculty of Biotechnology and Food Sciences, Wroclaw University of Environmental and Life Sciences, 37/41 Chełmońskiego Str., 51-630 Wroclaw, Poland; marzena.styczynska@upwr.edu.pl; 5 Department of Agricultural and Biosystems Engineering, Iowa State University, 4350 Elings Hall, Ames, IA 50011, USA; koziel@iastate.edu

**Keywords:** sewage sludge valorization, fuel properties, fertilizer, thermal treatment, torrefaction, nutrients, waste management, carbonized refuse-derived fuel, circular economy, zero waste

## Abstract

Sewage sludge (SS) recycling is an important part of the proposed ‘circular economy’ concept. SS can be valorized via torrefaction (also known as ‘low-temperature pyrolysis’ or ‘roasting’). SS can, therefore, be considered a low-quality fuel or a source of nutrients essential for plant growth. Biochar produced by torrefaction of SS is a form of carbonized fuel or fertilizer. In this research, for the first time, we tested the feasibility of torrefaction of SS with high ash content for either fuel or organic fertilizer production. The research was conducted in 18 variants (six torrefaction temperatures between 200~300 °C, and three process residence times of 20, 40, 60 min) in 5 repetitions. Fuel and fertilizer properties and multiple regression analysis of produced biochar were conducted. The higher heating value (HHV) of raw SS was 21.2 MJ·kg^−1^. Produced biochar was characterized by HHV up to 12.85 MJ·kg^−1^ and lower H/C and O/C molar ratio. Therefore, torrefaction of SS with high ash content should not be considered as a method for improving the fuel properties. Instead, the production of fertilizer appears to be favorable. The torrefaction increased C, N, Mg, Ca, K, Na concentration in relation to raw SS. No significant (*p* < 0.05) influence of the increase of temperature and residence time on the increase of biogenic elements in biochar was found, however the highest biogenic element content, were found in biochar produced for 60 min, under the temperature ranging from 200 to 240 °C. Obtained biochars met the Polish regulatory criteria for mineral-organic fertilizer. Therefore SS torrefaction may be considered a feasible waste recycling technology. The calculation of torrefaction energy and the mass balance shows energy demand <2.5 GJ∙Mg^−1^ w.m., and the expected mass yield of the product, organic fertilizer, is ~178 kg∙Mg^−1^ w.m of SS. Further investigation should consider the scaling-up of the SS torrefaction process, with the application of other types of SSs.

## 1. Introduction

Municipal sewage sludge (SS) is a ‘by-product’ produced during the municipal wastewater treatment process. It is estimated that the volume of generated SS constitutes about 2% of treated wastewater and contains more than half of the initial load of pollutants flowing with sewage to the treatment plant. Both the quantity and composition of the SS from wastewater treatment plants (WWTP) depends on the origin of treated sewage, the applied method of wastewater and sludge treatment, as well as the amount and properties of (e.g., industrial) sewage [1]. SS consists of ~55%–80% of water and up to 20%~45% of solids. The varied composition of SS from different sources and WWTPs processes implies a local, site-specific solution to the problem of SS disposal and utilization [2,3].

The main SS treatment methods are agricultural use, land reclamation, thermal treatment, and storage. SS management methods differ significantly between countries. Countries with high gross domestic product (GDP) prefer combustion as the primary method of processing, while countries with lower GDP are forced to landfilling. EU countries thermally treat ~22% of the total weight of the SS [4]. The most common method of SS treatment in Poland is agricultural usage (19%), followed by thermal treatment (14%) [5]. Between 2000 and 2015, Poland significantly increased the use of thermal methods and decreased the amount of landfilled SS and sludge stored in WWTP for future utilization. This trend is similar to treatment in countries with higher GDP than Poland’s, where the amount of thermally treated SS has significantly increased over the past years, and the amount of SS sent for storage, or landfilling has decreased [6]. Agricultural usage of SS is relatively inexpensive, and the mass of SS utilized in this manner in Poland has not increased significantly in recent years [5]. This is due to stricter requirements regulating agricultural sewage deposits and concerns about accumulated toxins in SS.

Direct agricultural applications of untreated SS may pose an environmental risk [7]. Therefore, SS pre-treatment methods are developed. One of the directions is thermochemical treatment. Thermochemical methods comprise many processes that lead to the utilization of a large amount of waste and create value-added products by reducing the volume of water by 80%~90% [8]. The main criteria to use thermal and thermochemical methods are the production of energy from locally available raw materials and the reduction of environmental contaminants and concentration of nutrients in biochar [9]. Combustion, pyrolysis, fast pyrolysis, slow pyrolysis, gasification [10], and torrefaction are in the center of thermochemical methods and lead to creating biochar [11,12]. Other thermal processes for biomass depolymerization are solvolysis [13,14,15]. All types of thermal conversion of municipal SS lead to the production of one or more ash char fractions. The quality and quantity of these products can vary and depends on the sludge’s source and the design of the thermal process. Thus, addressing the same question as in the manuscript title (fuel or fertilizer?) require further investigation and discussion on the suitability of other thermochemical conversion methods to produce either solid fuels or fertilizers.

Torrefaction is a biomass thermal decomposition process that produces a C-rich product—biochar [12,16,17,18,19,20,21,22,23,24,25]. Even though there is no exact definition and the term ‘biochar’ is loosely used for all char, which is thermochemically or hydrothermally produced out of biomass or waste products with high organic content, there are some limitations. Sometimes the term biochar is referring to material produced in temperature above 350 °C [26]. Other references are limiting the usage of the term depending on application or quality; for example, C content between 50% and 80% [27]. According to Lehmann et al. [28] and Sohi et al. [29], the term biochar implies the usage of the char for amelioration or C-sequestration. Polish regulations [30] prohibit the usage of terms ‘biocarbon’ and ‘toryfikat’ (from Polish: ‘torrefied biomass’) when it originated from SS. Considering that SS is regarded as ‘biomass’ in Polish legislation [30], the term ‘biochar’ will be used in this study.

During torrefaction, biomass partly decomposes, generating both condensable and non-condensable gases resulting in higher C content [28]. Torrefaction may also be referred to as ‘roasting,’ ‘slow and mild pyrolysis’, ‘wood-cooking’, and ‘high-temperature drying’ [31]. Apart from treated biomass, temperature and retention time are the main influencing factors on process efficiency [32]. The torrefaction is usually carried out in the temperature range between 200~300 °C, and the processing time of 15~20 min depending on the material properties [33]. Torrefaction is most commonly used for lignocellulosic biomass [34,35]. 

Torrefaction of SS has been initially characterized by Poudel et al. [36]. As demonstrated by research conducted by Poudel et al. [36], the SS is a suitable substrate for the torrefaction process. The increase in the C density was observed in the resulting product [36]. On the other hand, produced biochar contained a smaller amount of O and H in its structure. This was caused by the release of volatile compounds during the torrefaction included in the gaseous products. Poudel et al. [36] studies were conducted in material with low ash content. However, the high ash content in SS is site/source and treatment technology-specific. Other research on SS torrefaction was performed on materials characterized by ash content between 12 to 43%. Produced biochars calorific value ranged between 11.43 to 19.86 MJ∙kg^−1^ [36,37,38,39,40], with one research indicate extremely high devolatilization in the temperature range between 260~300 °C resulting in biochars with the lower heating value (LHV) from 6.50 to 3.88 MJ∙kg^−1^ [41].

Due to improved combustion properties, torrefaction products can be used as a fuel source for torrefaction of lignocellulosic biomass. In the case of SS, due to high degradability organic matter and relatively high ash content, torrefaction does not improve the fuel properties of SS [12] and can even decrease the calorific value [41]. Therefore, SS torrefaction may be considered as pre-treatment before SS biochar application in agriculture. 

Lignocellulosic biochar agricultural use is being researched in the context of biorenewable fuels. It has been proven that biochar application significantly improves soil quality and an essential C and other nutrients balance [42,43]. The positive effect of biochar on the soil is caused by the improvement of texture and porosity, which reflects changes in the physical, chemical and biological parameters. Nutrients in the biochar are more easily absorbed into their cell matrix. The ability to exchange cationic biochar, for example, allows the retention of K ions available to plants [44]. 

SS biochar produced in the pyrolysis process was characterized by a significant increase of K and P but increasing temperature above 300 °C caused rapid N loss via volatilization [45]. Yue et al. [46] tested pyrolysis SS biochar influence on poor quality urban soil. A positive effect of increased nutrient content in biochar co-composted with poultry manure was shown [47]. Addition of biochar from SS in the amount of 10 Mg∙ha^−1^ in a pot experiment, resulted in an increased yield of tomatoes by 64%, and a synergistic effect of increased availability of nutrients along with the improvement of soil quality. Biochar has a heterogeneous and highly porous structure. Both its internal and external surface is very extensive and has a different character both hydrophilic and hydrophobic. This makes biochar a material with high water retention capacity, which can be important for example, during the reclamation of poor soils [48].

Most publications on SS biochar agricultural properties is based on the pyrolysis process, that occurs in higher temperature, compared to torrefaction. Therefore, biochars generated from both processes will differ significantly. To date, the only research on the influence of torrefaction on the nutrient properties of SS biochar was published by Wesenbeeck et al. [49]. This is in contrast to a greater number of studies focused on the SS biochar agricultural application after pyrolysis [44,45,46,47,48]. Lessons learned from pyrolysis can be useful but are not necessarily applicable to SS torrefaction biochar.

This study aimed to evaluate the influence of torrefaction process residence time and temperature on essential fuel and fertilizer properties of SS with high initial ash content. The research was designed to perform analysis of both raw SS as reference samples as well as those generated in 18 variants of biochars (6 temperatures, 3 retention times). The scope of analyses included the characterization of fuel and fertilizer properties. The results of the analyses allowed us to develop multiple regression models describing the influence of torrefaction parameters on the properties of the generated torrefied product. Models can be used to identify torrefaction parameters with the highest efficiency and to propose the optimal use of the biochar as a fuel or fertilizer. The energy and mass balance of SS torrefaction was performed to analyze the applicability of this solution.

## 2. Materials and Methods

### 2.1. Sewage Sludge Characterization

Sewage sludge originated from municipal WWTP in Jastrzębie-Zdrój, Poland. The facility description and sample collection methods were described in Pulka et al. [41]. Raw SS properties used in this research are summarized in Table 1.

### 2.2. Experimental Design

Torrefaction, with the generation of biochar products, was performed using the method described in Białowiec et al. [50] in the muffle furnace 8.1/1100, SNOL, Utena, Lithuania. SS was dried for 24 h at 105 °C before the experiment in accordance with Stępień et al. [24]. The drying was because the dewatered (raw) SS had the moisture content of 80.2%. Thus, in order to avoid the influence of a long period of heat transfer to the sample, dry sludge was used with dry matter content of 96.3% (Table 1). Two independent factors were applied: torrefaction temperature and torrefaction duration. The target temperatures of 200, 220, 240, 260, 280, and 300 °C were maintained for 20-, 40- or 60-min. The reaction temperature was measured throughout the experiment. The total number of variants was 18. Each variant was performed in 5 repetitions.

The experimental procedure was the same as [24]. One crucible for a single torrefaction variant, contained between 250~300 g d.m. of SS, was put into the cold muffle furnace. CO_2_ was supplied at 10 dm^3^∙h^−1^ to ensure inert conditions during torrefaction. CO_2_ was introduced into the reactor by the steel 0.25-inch tube inserted through the muffle furnace chimney. The end of the tube was placed in the central point of the reactor chamber, above the crucible with the SS sample. CO_2_ and process gases were outflowing thought the reactor chimney. The heating of the reactor began 5 min after CO_2_ was introduced into the reactor. Heating always started at ambient temperature and took 5 to 10 min, depending on the target temperature. After torrefaction, the samples were left in the furnace to cool down for 60 to 90 min, depending on the initial process temperature. CO_2_ flow was shut off when the temperature inside the reactor dropped below 100 °C during cooling. The cooled sample was moved from the muffle and weighed with 0.1 mg accuracy to determine the mass loss. After that, the sample was analyzed. The cooling period was incorporated because the reactor could not be opened until the temperature drops down to a certain point. Without this procedure, we would expose the biochar to atmospheric oxygen and possible self-ignition could occur. 

### 2.3. Analyses of the Biochar Properties

Torrefied and dry SS samples were characterized by the analysis described below:

- Moisture content using the KBC65W (WAMED, Warsaw, Poland) laboratory dryer with Radwag PS 3500.R2 (Radwag, Radom, Poland) analytical balance following the PN-EN 14346:2011 standard [51],
- Losses on ignition (LOI) by means of model 8.1/1100, SNOL, Utena, Lithuania muffle furnace with Radwag PS 3500.R2 analytical balance following the PN-EN 15169:2011 standard [52],
- Ash content using the SNOL 8.1/1100 muffle furnace with Radwag PS 3500.R2 analytical balance following the PN-G-04516:1998 standard [53],
- Elementary C, H, N, and O composition using Perkin Elmer 2400 Series CHNS/O (Waltham, MA, USA) with Radwag, MYA 2.4 Y analyzer following PN-EN ISO 16948:2015-07 [54]
- HHV and LHV using the IKA C2000 Basic calorimeter (IKA® Poland Sp. z o.o., Warsaw, Poland) following the PN-G-04513:1981 standard [55],
- Mg, Ca, K, Na total content in solid samples were analyzed with atomic absorption spectroscopy (AAS) after dry mineralization using Varian Spektra AA 240 FS following PN-EN 14082: 2004 standard [56] (Agilent Technologies, Santa Clara, CA, USA). Dry mineralization was carried out with the procedure described below. The homogeneous sample (10 g) was incinerated on the heating plate; then the samples were mineralized in a muffle furnace for 8 h, the ash was burned for 2 h after dissolving in 2 cm^3^ HNO_3_. The mineralization was transferred quantitatively into 10 cm^3^ vessels using 2M HNO_3_.

### 2.4. Data Analysis

The following parameters were calculated based on the experiment results:

Mass yield,% [57,58]:

(1) $$\mathrm{My}=\left(\frac{M_{t}}{M_{0}}\right)\times 100$$

where M_0_ and M_t_ *is* mass before and after process, respectively, g.

Energy yield,% [57,58]:

(2) $$\mathrm{Ey}=\mathrm{My}\cdot \frac{{\mathrm{HHV}}_{t}}{{\mathrm{HHV}}_{o}}$$

where HHVt and HHVo is energy after and before the process, respectively, MJ·kg^−1^ d.m.

H/C ratio [36]:

(3) $$H/C=\frac{H/1}{C/12}$$

where H and C is the percentage of hydrogen and carbon content.

O/C ratio [36]:

(4) $$O/C=\frac{O/16}{C/12}$$

where O is the percentage of oxygen content.

The LHV was estimated based on HHV, moisture content (W), and the H content:

(5) LHV=HHV·22.42·(W+8.94·H)

where W is moisture content,%; 8.94 is the H-to-H_2_O conversion, H-hydrogen content,%

HHV_daf_ (on dry and on ash-free bases) was estimated as [59]:

(6) $${\mathrm{HHV}}_{\mathrm{daf}}=\frac{\mathrm{HHV}}{\mathrm{Mf}-\mathrm{Mash}}$$

where HHV—high heating value (on dry basis), MJ·kg^−1^; M_f_—fuel mass (on dry basis), mass, g; M_ash_—the mass of ash in fuel, g.

Multiple regression analysis at the significance level *p* < 0.05 was then performed to elucidate the influence of process temperature and residence time on biochars fuel and fertilizer properties. Finally, the polynomial models of process temperature and residence time influence on biochars fuel properties were used according to the previous study [60] with regression modeling:

(7) $$v=a1+a2\cdot T^{2}+a3\cdot T+a4\cdot t^{2}+a5\cdot t+a6\cdot T\cdot t+a7\cdot T^{2}\cdot t+a8\cdot T\cdot t^{2}+a9\cdot T^{2}\cdot t^{2}$$

where *v* is modeled dependent variable; *T* and *t* are independent variables of temperature and time, respectively; *a2–a9* are regression coefficients; *a1* is an intercept.

Statistica 13 software (StatSoft, Inc., TIBCO Software Inc., Palo Alto, CA, USA) was used to conduct statistical analysis. The detail regression coefficients values and statistical evaluation of determined coefficients describing the influence of torrefaction temperature and process duration on fuel and fertilizer parameters of SS biochars are given in Table A1, Table A2, Table A3, Table A4, Table A5, Table A6, Table A7, Table A8, Table A9, Table A10, Table A11, Table A12, Table A13, Table A14, Table A15, Table A16, Table A17 and Table A18 in the Appendix A. 

Torrefaction energy demand was calculated following [61]. Estimated price for covering the energy demand using pelletized biomass incineration was calculated with assumption (HHV = 20 MJ·kg^−1^, 1 Mg price = 999 PLN (1 USD ~ 4 PLN), incineration efficiency = 90%) [62]. Prices were given in EURO at the exchange rate from National Polish Bank in December 2019 (EURO = 4.261 PLN). The economic analysis of the biochar was based on the unit prices of mineral fertilizers used in Poland. For this purpose, the N fertilizer prices have been converted into the cost of 1 kg of a pure component following [63].

## 3. Results

### 3.1. The Influence of Torrefaction Temperature and Residence Time on Fuel Properties of Biochars

**Figure 1:**
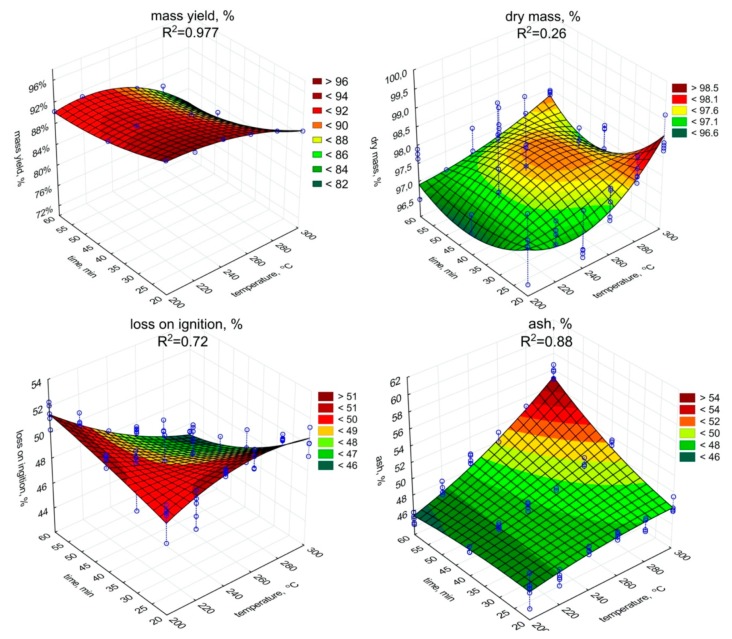
Mass yield, dry mass, loss on ignition, the ash content in torrefied SS in relation to process temperature, and residence time.

The significant (*p* < 0.05) decrease in the mass yield value with the increase of the temperature and retention time was observed (Figure 1, Table A1). The low moisture content in biochar samples was observed. It could be caused due to hygroscopic condensation of air vapor, and indirectly may indicate the degree of hydrophobicity of biochars. The dry matter content ranged from 97.4% to 98.5%. The increase in torrefaction temperature and retention time resulted in a significant (*p* < 0.05) dry matter increase (and decrease of water content) (Figure 1, Table A2). The lowest content of dry matter (97.4%) was observed in the biochar generated at 200 °C with a retention time of 20 min. Whereas the highest dry matter values were obtained for a retention time of 20 min at 300 °C (98.5%), and 97.8% at 300 °C with a retention time of 60 min. 

The LOI content in biochars (Figure 1) decreased due to the torrefaction in relation to raw SS (Table 1), while ash content (Figure 1) increased. The lowest LOI content, 44.4% d.m., was recorded for biochar produced during 60 min and under 300 °C, while the highest LOI content, 51.6% d.m., for 20 min of torrefaction under 200 °C. An opposite trend between LOI and ash content were observed. A significant (*p* < 0.05) increase in temperature and residence time resulted in LOI decrease during SS torrefaction (Figure 1, Table A3). The increase of temperature and retention time increased (*p* < 0.05) the ash content (Figure 1, Table A4). The highest ash content value 55.6% was obtained in the most robust variant of SS torrefaction (300 °C, 60 min). 

**Figure 2:**
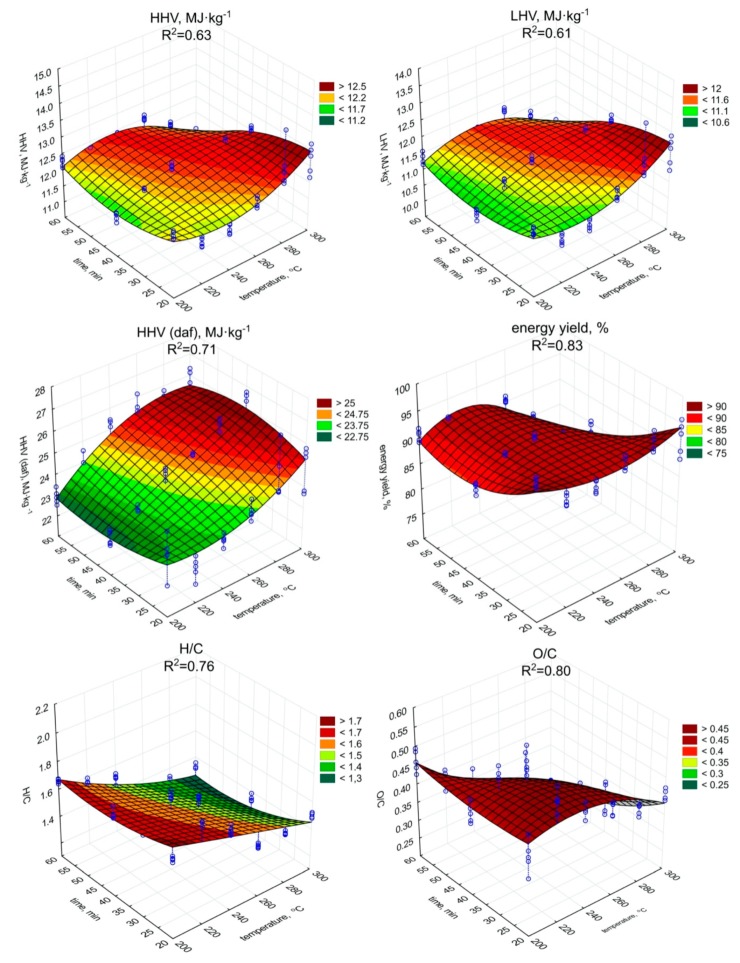
HHV, LHV, HHV daf., energy yield, and H/C, O/C molar ratios of torrefied SS in relation to process temperature, and residence time.

**Figure 3:**
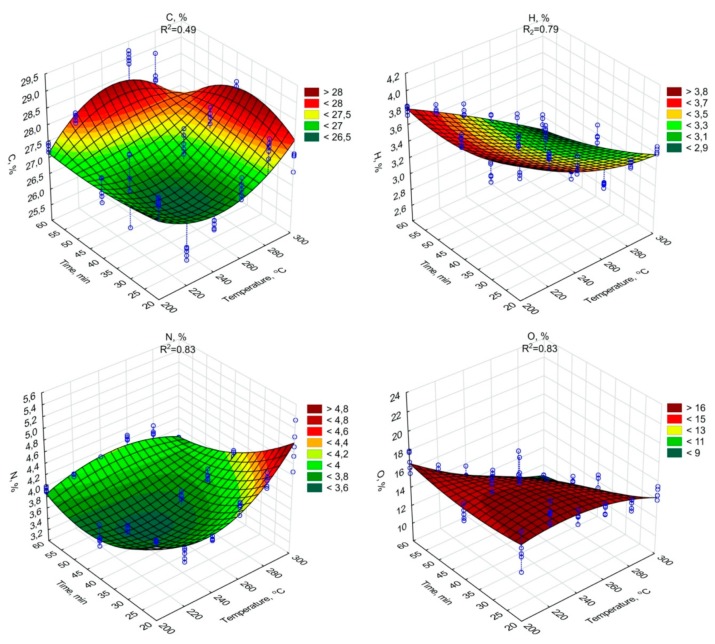
C, H, N, O percentage content in torrefied SS in relation to process temperature and residence time.

The torrefaction improved the fuel properties of the biochar (Figure 2 and Figure 3) in comparison to raw SS (Table 1), but only in individual cases. The highest HHV value was observed in the case of the biochar produced during the 60 min process under 260 °C (12.52 MJ·kg^−1^) (Figure 2). However, (surprisingly) the lowest observed HHV was found in the case of the biochar produced during the 60 min at 300 °C (11.09 MJ·kg^−1^). As the HHV depends on C content, the similar trend of the increase (*p* < 0.05) of HHV (Figure 2, Table A5), and C content in biochars (Figure 3, Table A6) with the temperature and residence time can be observed.

The highest C content, 29.02% d.m., (similarly as in the case of HHV), was found in biochar generated during 60 min of torrefaction under 260 °C (Figure 3). The increase of ash content with temperature and retention time did not negatively influence the HHV, while the C content in biochars increased significantly (*p* < 0.05). Additionally, the significant decrease of H (Figure 3, Table A7), and O (Figure 3, Table A8) content due to the increase in the temperature and duration of the torrefaction was observed. The H content was 3.74% d.m. in raw SS (Table 1). The lowest content of H 2.85% d.m. was in the case of 300 °C and 60 min (Figure 3). The H removal efficiency was 23.9%. The lowest O content was in the variant of 300 °C and 60 min (Figure 3). The removal efficiency in comparison to O content of 23.97% d.m. (Table 1) in raw SS was 64.9% in the case of the variant of 300 °C and 60 min (Figure 3). It shows that significant removal of O due to torrefaction offset the negative influence of the ash content increase on HHV values. However, the importance of the ash on fuel properties was demonstrated by the calculations of dry ash-free HHV daf. Simulation of ash removal made the trend of the significant (*p* < 0.05) increase in HHV daf with an increase in both temperature and residence time (Figure 2, Table A9) more apparent. The HHV daf. values were much higher than HHV. The highest HHV daf. was obtained for the SS torrefied at 300 °C, with a retention time of 40 min (26.2 MJ·kg^−1^).

The LHV is a function of HHV but additionally depends on moisture and H content. Although the changes in moisture content and H content in biochars obtained under different torrefaction conditions were statistically significant, they did not affect the pattern of LHV (Figure 2) depending both on process duration and temperature, which was similar to HHV. The LHV showed an upward trend (*p* < 0.05) as the time and temperature of the process increased (Figure 2, Table A10). The highest LHV (12.11 MJ·kg^−1^) value was associated with the biochar torrefied at 260 °C with 60 min residence time (Figure 2). Regression analyses indicated that temperature increase affects HHV and LHV values, causing an apparent rise when short residence times were used (Figure 2). For torrefaction above 20 min at higher temperatures, devolatilization was too robust, which is confirmed by significant (*p* < 0.05) decrease in LOI values, hence HHV and LHV values were declining. 

The energy yield showed a trend of significant (*p* < 0.05) decreasing value along with the increase of the process temperature (Figure 2, Table A11). At the retention time of 20 min, the percentage of energy yield ranged from 96.2% (200 °C) to 90.5% (240 °C). The results in the above retention time showed distinct fluctuation. With the 40 min process duration, the highest value was obtained at 240 °C (92.2%) and the lowest at 300 °C (84.5%). Two lowest results were obtained for biochar generated at the 60 min residence time at 280 and 300 °C with 84.2% and 73.3%, respectively. For the remaining lower process temperatures (200~260 °C), the highest value was obtained at 220 °C (91.5%). Regression analysis indicated significant (*p* < 0.05) energy yield drop at higher temperatures, due to substantial mass loss (Figure 1) despite the increase in HHV values (Figure 2).

**Figure 4:**
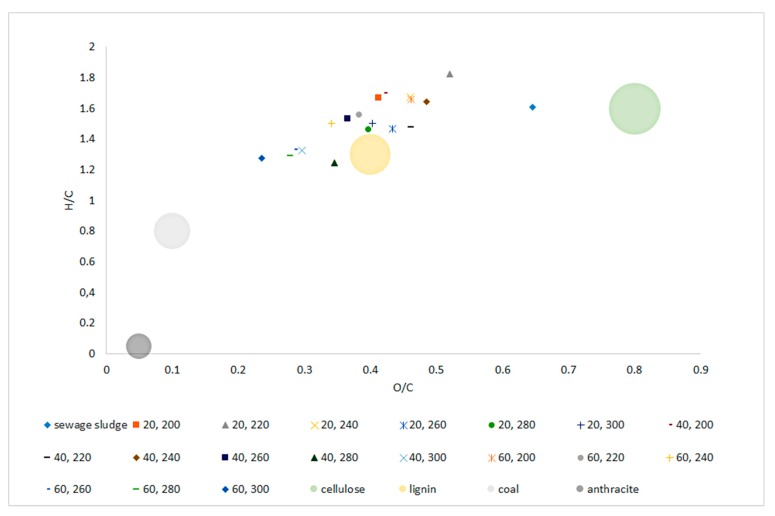
Van Krevelen diagram of dry sludge and torrefied sewage sludge generated at three retention times (20, 40, 60 min), in six temperature variants (200, 220, 240, 260, 280, 300 °C), in relation to coal (anthracite) and biomass (cellulose and lignin) reference.

The van Krevelen diagram (Figure 4) shows the material orientation relative to the molar O/C and H/C ratios. The tested SS was in the range characteristic for biomass between areas typical for cellulose and lignin. The apparent decrease in the ratio of both O/C and H/C, along with the increase in temperature and process retention time was significant (*p* < 0.05) and clearly visible (Figure 4, Table A12 and Table A13). Biochar samples H/C and O/C values obtained at 240~260 °C placed those materials in the area reserved for ‘lignin.’ Higher temperatures (280~300 °C) caused further decrease in H/C, O/C ratio, allowing biochars generated at those conditions to be placed outside of the ‘biomass area’, and trending into areas reserved for ‘coal.’ Regression models confirmed this trend indicating significant (*p* < 0.05) drops in H/C and O/C molar ratio with the increase of both temperature and process time with a high determination coefficient of 0.76 and 0.79, respectively.

### 3.2. The Influence of Torrefaction Temperature and Residence Time on Fertilizer Properties of Biochars

The N content in the raw SS was 4.3% d.m. (Table 1). The percentage of N content significantly (*p* < 0.05) increased with the process temperature (ranging from 3.4% to 4.9%) and was inversely proportional to the processing time (Figure 3, Table A14). 

**Figure 5:**
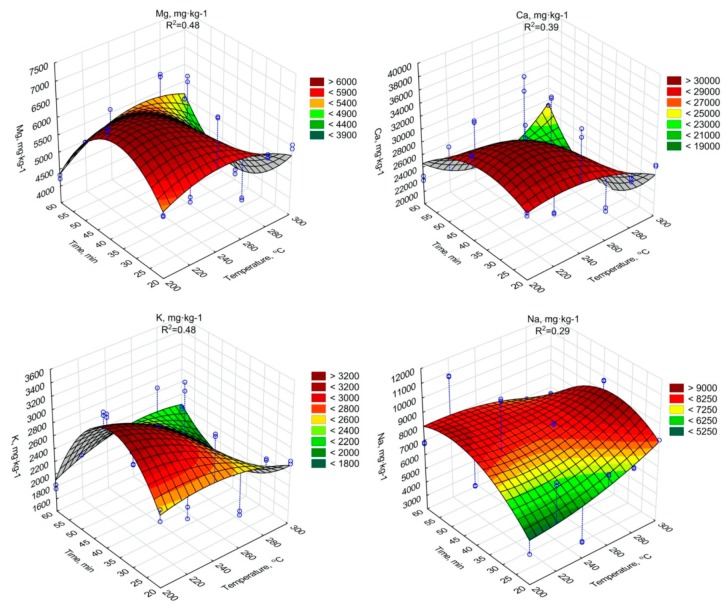
Mg, Ca, K, Na content in torrefied SS in relation to process residence time and temperature.

The regression analysis showed a significant decrease of the Mg, Ca, K content due to an increase of the torrefaction temperature and duration (Figure 5, Table A15, Table A16 and Table A17). In the case of Na similar tendency was confirmed, but with some fluctuations (Figure 5, Table A18). It should be noted, however, that all Mg, Ca, K, and Na concentrations in biochars were higher than in raw SS. It indicates that the torrefaction improved SS fertilizer properties, due to the densification of biogenic elements.

## 4. Discussion

The results were compared to biochars obtained from other SSs to discuss the differences or similarities obtained using similar feedstock and, in some cases, to lignocellulosic materials as a useful comparison showing that some of the observed trends are similar between vastly different substrates.

### 4.1. Proximate Analysis

SS dry matter values can be compared to a dry matter content ranging from 96.4% to 98.4% obtained in the work of Pulka et al. [12], or up to 99.8% described in the work of Atienza-Martinez et al. [64] and Dhungana [65]. The observed drop in the dry matter content, along with the increase of the process temperature may be related to the greater mass of condensates in the reactor during the cooling phase or be related to potentially higher hydrophobicity of the biochars obtained in higher temperatures.

The results of LOI were lower than in the work of Dhungana, [65]. This is due to the significantly lower initial volatile matter content of 57.2% d.m. compared to 76% d.m. reported by Dhungana [65]. In the work of Pulka et al. [12], the LOI results in biochars from SS obtained from the wastewater treatment plant in Olsztyn, Poland, ranged from 35.0% to 53.8% d.m., with an initial content of 54.4% d.m. The results obtained in this work are characterized by significantly lower LOI values, which may be related to a smaller torrefaction crucible; consequently, easier heat transfer into the sample. However, the trend of decrease in the LOI as the process temperature increases is apparent in all cited research including our recent study [41].

The ash content is the sum of the solid residue after the thermal decomposition of organic matter and non-flammable parts. A clear trend of ash content rise with the increase of temperature and the process duration is visible. This is due to the reduction of organic matter that is decomposed and degassed. Results of ash content in biochars obtained from SS originating from WWTP in Olsztyn presented in the work of Pulka et al. [12] ranging from 33% to 45% d.m. are lower relative to the results in this study. This indicates a significant impact of the source of SS on the obtained results and the economics of the possible implementation of SS torrefaction that needs to be site-specific. Generally, due to the high level of non-flammable fraction in SS, the use of torrefaction for SS processing should be counted with the increase of ash content during processing, which adversely affects fuel parameters. It also has a very unfavorable impact on the mass balance of the further thermal conversion of the obtained biochars, because a significant part of the mass (ash) will remain after the process as a waste requiring further utilization.

### 4.2. Fuel Properties

Higher heating value is a parameter that indicates the amount of energy that can be generated from the mass of the substrate during combustion, including the energy generated during gas condensation [66]. The HHV results obtained indicate that the torrefaction of the tested SS in more than 20 min may result in lower HHV values. Confirmation of this theory is in the results described elsewhere [12,41,50], where the torrefaction of two non-lignocellulosic materials, SS and RDF respectively, in 60 min residence time, did not result in statistically significant higher HHV values in relation to the substrate. In the work of Poudel et al. [36], a similar relationship occurred. The increase in the duration of the process above 30 min for the tested temperatures of 250 and 300 °C caused a decrease in the HHV in relation to the values obtained in shorter process times. A similar trend was visible in the work of Atienza-Martinez et al. [64], where lengthening of the process resulted in lowering the product HHV. Obtained HHV results are in line with the influence of temperature increase and residence time on the reduction of organic matter content (LOI). The HHV did not decrease as the organic C content increased (mainly compounds with high H and O concentration decomposed and degassed). The opposite tendency in comparison with lignocellulosic biomass may result from lower torrefaction activation energy of SS [12,41] and, consequently, the higher susceptibility of organic substances contained in SS to decomposition by degassing than organic substances from wood biomass.

The LHV increased significantly in relation to the raw SS and the dry SS. Such an increase in calorific value is associated with evaporation of 95% of water. For comparison, in research on SS torrefaction from the wastewater treatment plant in Olsztyn [12], the LHV increased from 0.5 MJ·kg^−1^ to 14.4 MJ·kg^−1^ generated at 200 °C. There was also an increase in the calorific value in relation to the dry SS. The maximum LHV value in the present study was 7% higher than the values characterizing dry SS. Such a small increase in LHV is associated with increased ash content, hence lowering volatile matter percentage in the biochars in relation to the dry SS. 

The parameter allowing to assess the impact of the valorization process on energy value in relation to the organic matter is the HHV daf. (dry ash-free). There was a visible trend of increasing calorific value together with the increase of temperature and duration of the process. A similar tendency was confirmed in the work of Atienza-Martinez et al. [64], where the increase in both retention time and temperature resulted in higher HHV daf. values for biochars. In the work of Pulka et al. [12], temperature rise caused a similar effect.

The energy yield is a parameter strongly correlated with the percentage mass yield. As in the case of mass yield, the decrease in the energy yield is associated with both the increase of the process temperature and the increase of the retention time. It should be noted that the energy yield dropped below 80% only with the longest retention time and the highest process temperature. This is likely caused by a relatively small decrease in mass and an increase in the HHV. Much higher residual energy values were obtained compared with the biochars obtained from SS by Dhungana [65]. The results in Poudel et al. [36], where the values obtained at 300 °C amounted to approx. 55%. It is related to the lower initial percentage of volatile content in SS used in this research, compared to the works of Dhungana [65] and Poudel et al. [36]. Similar values of residual energy for SS were observed in Atienza-Martinez et al. [64].

The content of C in the biochar increased with the increase in temperature and process residence time. A similar trend is visible for the previous work done by Pulka et al. [12]. It should be noted, however, that the increases in the percentage of C are lower than in the cited publication. 

An opposite trend was observed for the H and O content in biochars. The H and O content decreased as the temperature and duration of the process increased. A similar trend was reported in the works of Mock et al. [67] and Pulka et al. [12]. In both cases, the H content decreases were observed as the temperature increased, and also with the increase of the retention time in the case of Mock et al. [67]. It is worth emphasizing that in the work of Mock et al. [67], much higher H concentrations were observed, which may be associated with almost 2× higher content of this element in the raw material than in the presented studies. The decrease in O content is most strongly associated with degassing, in particular, CO_2_, CO, and O, and VOCs containing carboxyl, carbonyl, and hydroxyl groups. Confirmation of the described trend is reported, among others, in [12,36], where the increase in temperature caused a significant reduction in the O content in the processed SS. A similar trend, but not as apparent, is visible in the work of Huang et al. [68], where despite the higher range values of the analyzed temperatures, the decreases in O content were not as high.

Van Krevelen diagram allows the material to be positioned in relation to O/C and H/C molar ratios, allows for quick determination of material potential when considering thermal processing. Increasing the temperature of the SS torrefaction process resulted in a decrease in H/C and O/C ratios, and thus a shift in biochars positions towards higher calorific materials. The raw SS was located in the area characteristic for cellulose, while the biochars were located in the area characteristic for lignin’s and were approaching the area reserved for hard coal. Such shaping of the van Krevelen diagram along with the increase in the temperature and retention time of torrefaction is typical and confirmed in the works of others [36,69].

### 4.3. Fertilizer Properties

Similarly, to this research, the increase in N content with increasing temperature can be observed in Poudel et al. [36]. However, it should be noted that the relative increase in N content in the cited work was significantly lower. The results obtained for the biochars in Huang et al. [68] were different, due to a decrease in N content with the rise of the process temperature.

The increase of other biogenic elements Mg, Ca, K, and Na with the increase of the temperature and torrefaction residence time was observed. The increase of Mg content during the SS torrefaction process at 300 °C was obtained in the work of Lu et al. [70], where increase occurred compared to three different SS samples from 4.1 to 8.1 g·kg^−1^ d.m., from 6.7 to 11.6 g·kg^−1^ d.m. and from 4.1 to 5.4 g·kg^−1^ d.m. A similar trend, although with lower growths, was presented by Hossain et al. [71], where the Mg content increased from 3.3 to 3.5 g·kg^−1^ d.m. In the case of Ca, Hossain et al. [71] observed that Ca content increased from 30.2 to 34.7 g·kg^−1^ d.m., while in the work of Lu et al. [70] where values for 3 different SS sample increased from 4.8 to 8.1 g·kg^-1^ d.m., 6.7 to 11.6 g·kg^−1^ d.m. and from 1.5 to 1.8 g·kg^−1^ d.m. The changes of K content in biochars had similar character as results obtained in the work of Lu et al. [70], where the K content in biochars from 3 different samples of dry SS ranged from 1.2 to 2.1 g·kg^−1^ d.m., 0.8 to 1.6 g·kg^−1^ d.m. and 1.3 to 1.8 g·kg^−1^ d.m. Additionally, in the case of Na, Lu et al. [70] showed a similar tendency indicating an increase in the value of generated SS biochars in relation to the dry sludge.

The obtained results for N, Ca, Mg, K, Na content indicate that torrefaction of SS can be considered as a method of improving the SS fertilizing properties, and thus enabling its recycling in agriculture. The amount of organic matter, the content of total N and K (as K_2_O), allows qualifying of the obtained biochars as solid mineral-organic fertilizers following Polish regulations [72]. Examples of effective usage of biochar from SS as fertilizers for agriculture are described in the works of Waqas et al. [73], Hossain et al. [74], Song et al. [75], and Zornoza et al. [76], where the biochars were generated from SS under the pyrolytic conditions, and their high agricultural suitability was found. However, the manuscript did not address the question concerning which of the direction of the biochars from SS utilization (fuel or fertilizer) is more preferable.

Hossain et al. [74] verified the hypothesis about the agro-technical properties of pyrolyzed biochar in relation to the improvement of soil quality, plant growth, yield, and slight bioavailability of metals in tomatoes. The results showed an improvement in vegetable growth by 64% as a result of the increased availability of P and N, as well as the improvement of chemical soil conditions. The yield reached its maximum following the application of char with fertilizer. Shinogi and Kanri [77] reported other positive aspects of biochar from animal and human waste pyrolysis applications to the soil. It was noted that due to its lightness, it is a potential material for improving water permeability. However, the authors pointed to the need for monitoring the changes in the content of Zn in the biochars obtained in the low-temperature pyrolysis processes. The varied impact of biochar on the development of indicator organisms was caused by the inhomogeneous composition of waste sent to the reactor.

The simplified economic evaluation of the SS torrefaction for fertilization purposes was added as the results indicate that biochar produced from the SS has a higher potential for application in agriculture than for energy purposes. The energy demand of 1 Mg of raw SS drying and torrefaction amounted to 2.43 GJ, with only 2% of the overall value was needed for torrefaction purposes. Most of the energy was required for water evaporation during the heating stage of the process. The price of energy necessary for raw SS torrefaction was calculated using the pelletized biomass incineration process and amounted for 32 EURO∙Mg^−1^ of raw SS. Taking into account the percentage of N in the torrefied samples (240 °C, 1 h), the price of N considered as pure fertilizer was evaluated. The analysis indicates that 178.4 kg of biochar is produced from 1 Mg of raw SS, with a fertilization value of 5 EURO. It is worth mentioning that with decreasing moisture, less energy will be required for drying and torrefaction.

Taking into account that torrefaction did significantly increase N, Ca, Mg, K, and Na compounds content torrefied SS and that many researchers pointed out that SS biochars had a positive influence on plant growth, this mode of SS processing appears to be favored. Considering this research as an initial study, further evaluation of torrefaction biochar from SS for agricultural purposes should be conducted. Future research should include bioavailability and leachability of biogenic compounds, the influence of the SS biochar on soil texture and porosity, as well as availability of cationic part to the plants. Next, the biochar fertilization field tests and toxicological assessment should be conducted.

## 5. Conclusions

In this research, for the first time, we tested the feasibility of torrefaction of SS with high ash content for either fuel or organic fertilizer production. This research indicated that a better solution is to consider the biochar produced from torrefied SS as a fertilizer. The concentration of biogenic compounds increased as a result of SS torrefaction. The obtained biochars may be classified as a solid mineral-organic fertilizer according to the Polish regulations. The best biochars (i.e., with the highest biogenic element content, were the biochars produced from 200 to 240 °C and 60 min. Considering the high agricultural potential of SS biochar from the torrefaction process in this initial research, future tests including bioavailability and leachability of biogenic compounds and heavy metals, biochar fertilization field tests, and toxicological assays should be conducted. Additionally, the influence of SS biochar on soil physical properties should be studied. The selected fuel properties are also worth highlighting. Particularly interesting are the results of HHV and H/C and O/C molar ratios, demonstrating a slight improvement of the biochar fuel parameters. Unfortunately, the increase in the ash content along with the increase in temperature and the retention time-limited the feasibility of further improvement to fuel parameters. The best variant of SS torrefaction (from the fuel as an end-use scenario) were biochars generated at 280 °C and 20 min. The increase of the H/C and O/C ratios, with a slight increase in the ash content, resulting in high values of both the HHV and the LHV was observed along with the increase of the torrefaction temperature. Obtained results indicate that torrefaction of SS with high ash content did not increase fuel properties (especially HHV and LHV). Additionally, the biochars were characterized by much higher ash content than the raw SS.

## Figures and Tables

**Table 1:** Raw sewage sludge properties.

Property	Value
dry mass,%	20.3
loss on ignition,% d.m.	57.2
ash,% d.m.	38.5
LHV, MJ·kg^−1^	0.4
HHV, MJ·kg^−1^	12.2
HHV daf. MJ·kg^−1^	20.6
C,% d.m.	27.9
H,% d.m.	3.7
N,% d.m.	4.3
S,% d.m.	1.6
O,% d.m.	23.9
H/C ratio	1.6
O/C ratio	0.6
Mg, mg·kg^−^^1^, d.m.	2,643
Ca, mg·kg^−^^1^, d.m.	14,640
K, mg·kg^−^^1^, d.m.	1535
Na, mg·kg^−^^1^, d.m.	3511

Note: LHV—lower heating value; HHV—higher heating value; HHV daf.—dry ash-free higher heating value (on dry and on ash-free bases).

## Appendix A

**Table A1:** The statistical parameters of polynomial regression analysis of the influence of SS torrefaction temperature and residence time on process mass yield.

Mass Yield, %						
Model	Mass Yield, % = (1.44809) + (7.03892 × 10^-6^)·T^2^ + (−0.00364092)·T + (0.000414299)·t^2^+ (−0.0460416)·t + (0.000374584)·T·t + (−8.04432 × 10^-7^)·T^2^·t + (−3.39151 × 10^−6^)·T·t^2^ + (7.27706 × 10^−9^)·T^2^·t^2^ R^2^ = 0.98					
Function Parameters	Value	Standard Error	t Value	*p* Value	Lower Confidence Limit	Upper Confidence Limit
a1	1.448091	0.285892	0.00	0.00	0.879256	2.016927
a2	0.000007	0.000000	0.00	0.00	0.000007	0.000007
a3	−0.003641	0.002318	0.00	0.00	−0.008253	0.000971
a4	0.000414	0.000201	0.00	0.00	0.000015	0.000814
a5	−0.046042	0.016232	0.00	0.00	−0.078339	−0.013745
a6	0.000375	0.000132	0.00	0.00	0.000113	0.000636
a7	−0.000001	0.000000	0.00	0.00	−0.000001	−0.000001
a8	−0.000003	0.000000	0.00	0.00	−0.000003	−0.000003
a9	0.000000	0.000000	0.00	0.00	0.000000	0.000000

**Table A2:** The statistical parameters of polynomial regression analysis of the influence of SS torrefaction temperature and residence time on dry mass content in biochars.

dry mass, %						
Model	d.m., % = (223.1) + (0.002)·T^2^ + (−1.053)·T + (0.083)·t^2^ + (−7.035)·t + (0.0583)·T·t + (−0.0001)·T^2^· t + (−0.0007)·T·t^2^ + (1.393 × 10^−6^)·T^2^·t^2^ R^2^ = 0.26					
Function Parameters	value	Standard Error	t Value	*p* Value	Lower Confidence Limit	Upper Confidence Limit
a1	223.1321	39.68104	0.00	0.00	144.1793	302.0849
a2	0.0022	0.00064	0.00	0.00	0.0009	0.0034
a3	−1.0528	0.32172	0.00	0.00	−1.6929	−0.4127
a4	0.0828	0.02787	0.00	0.00	0.0273	0.1382
a5	−7.0352	2.25299	0.00	0.00	−11.5179	−2.5524
a6	0.0583	0.01827	0.00	0.00	0.0219	0.0946
a7	−0.0001	0.00004	0.00	0.00	−0.0002	−0.0000
a8	−0.0007	0.00023	0.00	0.00	−0.0011	−0.0002
a9	0.0000	0.00000	0.00	0.00	0.0000	0.0000

**Table A3:** The statistical parameters of polynomial regression analysis of the influence of SS torrefaction temperature and residence time on loss on ignition content in biochars.

Loss on Ignition, % d.m.						
Model	LOI, % = (−43.517) + (−0.0012353)·T^2^ + (0.702014)·t + (−0.0291921)·T^2^ + (3.83877)·T + (−0.0285369)·t·T + (4.89634 × 10^−5^)·t^2^·T + (0.000221984)·T·t^2^ + (−3.90553 × 10^−7^)·T^2^·t^2^ R^2^ = 0.72					
Function Parameters	Value	Standard Error	t Value	*p* Value	Lower Confidence Limit	Upper Confidence Limit
a1	−43.5170	58.73534	0.00	0.00	−160.382	73.34794
a2	−0.0012	0.00095	0.00	0.00	−0.003	0.00066
a3	0.7020	0.47621	0.00	0.00	−0.245	1.64952
a4	−0.0292	0.04126	0.00	0.00	−0.111	0.05290
a5	3.8388	3.33485	0.00	0.00	−2.797	10.47407
a6	−0.0285	0.02704	0.00	0.00	−0.082	0.02526
a7	0.0000	0.00005	0.00	0.00	−0.000	0.00016
a8	0.0002	0.00033	0.00	0.00	−0.000	0.00089
a9	−0.0000	0.00000	0.00	0.00	−0.000	−0.00000

**Table A4:** The statistical parameters of polynomial regression analysis of the influence of SS torrefaction temperature and residence time on ash content in biochars.

Ash Content, % d.m.						
Model	ash, % = (14.0912) + (−0.000486683)·T^2^ + (0.256399)·t + (−0.00923204)·t^2^ + (1.13906)·t + (−0.00882318)·T·t + (1.67301 × 10^−5^)·T^2^·t + (4.9036 × 10^−5^)·t·T^2^ + (−3.61091 × 10^−8^)·t^2^·T^2^ R^2^ = 0.88					
Function Parameters	Value	Standard Error	t Value	*p* Value	Lower Confidence Limit	Upper Confidence Limit
a1	14.09118	43.69689	0.00	0.00	−72.8519	101.0343
a2	−0.00049	0.00071	0.00	0.00	−0.0019	0.0009
a3	0.25640	0.35428	0.00	0.00	−0.4485	0.9613
a4	−0.00923	0.03069	0.00	0.00	−0.0703	0.0518
a5	1.13906	2.48099	0.00	0.00	−3.7973	6.0755
a6	−0.00882	0.02012	0.00	0.00	−0.0488	0.0312
a7	0.00002	0.00004	0.00	0.00	−0.0001	0.0001
a8	0.00005	0.00025	0.00	0.00	−0.0004	0.0005
a9	−0.00000	0.00000	0.00	0.00	−0.0000	−0.0000

**Table A5:** The statistical parameters of polynomial regression analysis of the influence of SS torrefaction temperature and residence time on HHV of the biochars.

HHV, MJ·kg^−1^						
Model	HHV, MJ·kg^−1^ = (56.7993) + (0.000704123)·T^2^ + (−0.35836)·T + (0.0154298)·t^2^ + (−1.93107)·t + (0.0147992)·T·t + (−2.75086 × 10^−5^)·T^2^·t + (−0.000109331)·T·t^2^ + (1.82382 × 10^−7^)·T^2^·t^2^ R^2^ = 0.63					
Function Parameters	Value	Standard Error	t Value	*p* Value	Lower Confidence Limit	Upper Confidence Limit
a1	56.79931	14.38158	0.00	0.00	28.18447	85.41415
a2	0.00070	0.00023	0.00	0.00	0.00024	0.00117
a3	−0.35836	0.11660	0.00	0.00	−0.59036	−0.12636
a4	0.01543	0.01010	0.00	0.00	−0.00467	0.03553
a5	−1.93107	0.81655	0.00	0.00	−3.55574	−0.30640
a6	0.01480	0.00662	0.00	0.00	0.00163	0.02797
a7	−0.00003	0.00001	0.00	0.00	−0.00005	−0.00000
a8	−0.00011	0.00008	0.00	0.00	−0.00027	0.00005
a9	0.00000	0.00000	0.00	0.00	0.00000	0.00000

**Table A6:** The statistical parameters of polynomial regression analysis of the influence of SS torrefaction temperature and residence time on the C content in biochars.

C, % d.m.						
Model	C, % = (−6.51131) + (−0.000660336)·T^2^ + (0.304374)·T + (−0.0668472)·t^2^ + (4.04284)·t + (−0.0353957)·T·t + (7.50162 × 10^−5^)·T^2^·t + (0.000579037)·T·t^2^ + (−1.21488 × 10^−6^)·T^2^·t^2^ R^2^ = 0.49					
Function Parameters	Value	Standard Error	t Value	*p* Value	Lower Confidence Limit	Upper Confidence Limit
a1	−6.51131	30.69734	0.00	0.00	−67.5894	54.56676
a2	−0.00066	0.00050	0.00	0.00	−0.0016	0.00033
a3	0.30437	0.24889	0.00	0.00	−0.1908	0.79958
a4	−0.06685	0.02156	0.00	0.00	−0.1098	−0.02394
a5	4.04284	1.74291	0.00	0.00	0.5750	7.51068
a6	−0.03540	0.01413	0.00	0.00	−0.0635	−0.00728
a7	0.00008	0.00003	0.00	0.00	0.0000	0.00013
a8	0.00058	0.00017	0.00	0.00	0.0002	0.00093
a9	−0.00000	0.00000	0.00	0.00	−0.0000	−0.00000

**Table A7:** The statistical parameters of polynomial regression analysis of the influence of SS torrefaction temperature and residence time on the H content in biochars.

H, % d.m.						
Model	H, % = (11.1242) + (8.72369 × 10^−5^)·T^2^ + (−0.0504356)·T + (0.00103414)·t^2^ + (−0.162667)·t + (0.00102235)·T·t + (−1.89313 ×·10^−6^)·T^2^·t + (−4.26597 × 10^−6^)·T·t^2^ + (4.46763 × 10^−9^)·T^2^·t^2^ R^2^ = 0.79					
Function Parameters	Value	Standard Error	t Value	*p* Value	Lower Confidence Limit	Upper Confidence Limit
a1	11.12415	7.851332	0.00	0.00	−4.49753	26.74584
a2	0.00009	0.000127	0.00	0.00	−0.00017	0.00034
a3	−0.05044	0.063657	0.00	0.00	−0.17709	0.07622
a4	0.00103	0.005515	0.00	0.00	−0.00994	0.01201
a5	−0.16267	0.445779	0.00	0.00	−1.04963	0.72429
a6	0.00102	0.003614	0.00	0.00	−0.00617	0.00821
a7	−0.00000	0.000000	0.00	0.00	−0.00000	−0.00000
a8	−0.00000	0.000045	0.00	0.00	−0.00009	0.00008
a9	0.00000	0.000000	0.00	0.00	0.00000	0.00000

**Table A8:** The statistical parameters of polynomial regression analysis of the influence of SS torrefaction temperature and residence time on the O content in biochars.

O, % d.m.						
Model	O, % = (94.3211) + (0.00132001)·T^2^ + (−0.646472)·T + (0.110223)·t^2^ + (−7.32599)·t + (0.0612511)·T·t + (−0.000125945)·T^2^·t + (−0.000907679)·T·t^2^ + (1.82155 × 10^−6^)·T^2^·t^2^ R^2^ = 0.83					
Function Parameters	Value	Standard Error	t Value	*p* Value	Lower Confidence Limit	Upper Confidence Limit
a1	1051.470	55631.35	0.02	0.99	−113095	115197.6
a2	0.248	0.90	0.27	0.79	−2	2.1
a3	−47.137	450.67	−0.105	0.92	−972	877.6
a4	5.535	38.52	0.14	0.88	−74	84.6
a5	−538.670	3116.91	−0.17	0.86	−6934	5856.7
a6	10.229	25.24	0.40	0.69	−42	62.0
a7	−0.031	0.05	−0.61	0.54	−0	0.1
a8	−0.122	0.31	−0.39	0.70	−1	0.5
a9	0.000	0.00	0.61	0.5	−0	0.0

**Table A9:** The statistical parameters of polynomial regression analysis of the influence of SS torrefaction temperature and residence time on HHV daf of the biochars.

HHV (daf), MJ·kg^−1^						
Model	HHV (daf), MJ·kg^−1^ = (64.4828) + (0.000608875)·T^2^ + (−0.319667)·T + (0.00555782)·t^2^ + (−1.47697)·t + (0.0100922)·T·t + (−1.58506 × 10^−5^)·T^2^·t + (−2.18888 × 10^−5^)·T·t^2^ + (−5.1846 × 10^−9^)·T^2^·t^2^ R^2^ = 0.71					
Function Parameters	Value	Standard Error	t Value	*p* Value	Lower Confidence Limit	Upper Confidence Limit
a1	64.48283	32.93082	0.00	0.00	−1.03918	130.0048
a2	0.00061	0.00053	0.00	0.00	−0.00045	0.0017
a3	−0.31967	0.26700	0.00	0.00	−0.85090	0.2116
a4	0.00556	0.02313	0.00	0.00	−0.04047	0.0516
a5	−1.47697	1.86972	0.00	0.00	−5.19713	2.2432
a6	0.01009	0.01516	0.00	0.00	−0.02007	0.0403
a7	−0.00002	0.00003	0.00	0.00	−0.00008	0.0000
a8	−0.00002	0.00019	0.00	0.00	−0.00040	0.0004
a9	−0.00000	0.00000	0.00	0.00	−0.00000	−0.0000

**Table A10:** The statistical parameters of polynomial regression analysis of the influence of SS torrefaction temperature and residence time on LHV of the biochars.

LHV, MJ·kg^−1^						
Model	LHV, MJ·kg^−1^ = (57.3765) + (0.000737877)·T^2^ + (−0.373049)·T + (0.0172241)·t^2^ + (−2.06729)·t + (0.015999)·T·t + (−2.99897 × 10^−5^)·T^2^·t + (−0.000125133)·T·t^2^ + (2.15415 × 10^−7^)·T^2^·t^2^ R^2^ = 0.61					
Function Parameters	Value	Standard Error	t Value	*p* Value	Lower Confidence Limit	Upper Confidence Limit
a1	57.37645	14.96197	0.00	0.00	27.60682	87.14608
a2	0.00074	0.00024	0.00	0.00	0.00026	0.00122
a3	−0.37305	0.12131	0.00	0.00	−0.61441	−0.13168
a4	0.01722	0.01051	0.00	0.00	−0.00369	0.03814
a5	−2.06729	0.84950	0.00	0.00	−3.75753	−0.37704
a6	0.01600	0.00689	0.00	0.00	0.00229	0.02970
a7	−0.00003	0.00001	0.00	0.00	−0.00006	−0.00000
a8	−0.00013	0.00009	0.00	0.00	−0.00029	0.00004
a9	0.00000	0.00000	0.00	0.00	0.00000	0.00000

**Table A11:** The statistical parameters of polynomial regression analysis of the influence of SS torrefaction temperature and residence time on the energy yield of the process.

Energy Yield, %						
Model	energy yield, % = (500.253) + (0.00637136)·T^2^ + (−3.23363)·T + (0.179189)·t^2^ + (−20.5477)·t + (0.161123)·T·t + (−0.00031386)·T^2^·t + (−0.00135047)·T·t^2^ + (2.50551 × 10^−6^)·T^2^·t^2^ R^2^ = 0.83					
Function Parameters	Value	Standard Error	t Value	*p* Value	Lower Confidence Limit	Upper Confidence Limit
a1	500.2532	109.4272	0.00	0.00	282.5274	717.9791
a2	0.0064	0.0018	0.00	0.00	0.0028	0.0099
a3	−3.2336	0.8872	0.00	0.00	−4.9989	−1.4684
a4	0.1792	0.0769	0.00	0.00	0.0262	0.3321
a5	−20.5477	6.2130	0.00	0.00	−32.9097	−8.1858
a6	0.1611	0.0504	0.00	0.00	0.0609	0.2614
a7	−0.0003	0.0001	0.00	0.00	−0.0005	−0.0001
a8	−0.0014	0.0006	0.00	0.00	−0.0026	−0.0001
a9	0.0000	0.0000	0.00	0.00	0.0000	0.0000

**Table A12:** The statistical parameters of polynomial regression analysis of the influence of SS torrefaction temperature and residence time on the H/C ratio in biochars.

H/C						
Model	H/C = (6.15786) + (6.41009 × 10^−5^)·T^2^ + (−0.0338503)·T + (0.00373692)·t^2^ + (−0.263384)·t + (0.0021366)·T·t + (−4.4153 × 10^−6^)·T^2^·t + (−3.03887 × 10^−5^)·T·t^2^ + (6.18517 × 10^−8^)·T^2^·t^2^ R^2^ = 0.76					
Function Parameters	Value	Standard Error	t Value	*p* Value	Lower Confidence Limit	Upper Confidence Limit
a1	6.1579	4.1683	0	0	−2.358	14.4515
a2	0.0001	0.0001	0	0	−0.0001	0.0002
a3	−0.0339	0.0338	0	0	−0.1011	0.0334
a4	0.0037	0.0029	0	0	−0.0021	0.0096
a5	−0.2634	0.2367	0	0	−0.7343	0.2075
a6	0.0021	0.0019	0	0	−0.0017	0.0060
a7	0.0000	0.0000	0	0	0.0000	0.0000
a8	0.0000	0.0000	0	0	−0.0001	0.0000
a9	0.0000	0.0000	0	0	0.0000	0.0000

**Table A13:** The statistical parameters of polynomial regression analysis of the influence of SS torrefaction temperature and residence time on the O/C ratio in biochars.

O/C						
Model	O/C = (2.85356) + (4.1663 × 10^−5^)·T^2^ + (−0.0202011)·T + (0.0038009)·t^2^ + (−0.246543)·t + (0.00208228)·T·t + (−4.3088 × 10^−6^)·T^2^·t + (−3.16778 × 10^−7^)·T·t^2^ + (6.4223 × 10^−8^)·T^2^·t^2^ R^2^ = 0.80					
Function Parameters	Value	Standard Error	t Value	*p* Value	Lower Confidence Limit	Upper Confidence Limit
a1	2.8536	1.8909	0	0	−0.9087	6.6159
a2	0.0000	0.0000	0	0	0.0000	0.0001
a3	−0.0202	0.0153	0	0	−0.0507	0.0103
a4	0.0038	0.0013	0	0	0.0012	0.0064
a5	−0.2465	0.1074	0	0	−0.4602	−0.0329
a6	0.0021	0.0009	0	0	0.0004	0.0038
a7	0.0000	0.0000	0	0	0.0000	0.0000
a8	0.0000	0.0000	0	0	−0.0001	0.0000
a9	0.0000	0.0000	0	0	0.0000	0.0000

**Table A14:** The statistical parameters of polynomial regression analysis of the influence of SS torrefaction temperature and residence time on the N content in biochars.

N, % d.m.						
Model	N, % = (13.737) + (0.000154911)·T^2^ + (−0.0716041)·T + (−0.00987428)·t^2^ + (0.322928)·t + (−0.00346963)·T·t + (7.1607×10^−6^)·T^2^·t + (9.22803 × 10^−5^)·T·t^2^ + (−1.91518 × 10^−7^)·T^2^·t^2^ R^2^ = 0.83					
Function Parameters	Value	Standard Error	t Value	*p* Value	Lower Confidence Limit	Upper Confidence Limit
a1	13.73703	7.673398	0.00	0.00	−1.53063	29.00468
a2	0.00015	0.000124	0.00	0.00	−0.00009	0.00040
a3	−0.07160	0.062214	0.00	0.00	−0.19539	0.05218
a4	−0.00987	0.005390	0.00	0.00	−0.02060	0.00085
a5	0.32293	0.435677	0.00	0.00	−0.54393	1.18979
a6	−0.00347	0.003532	0.00	0.00	−0.01050	0.00356
a7	0.00001	0.000000	0.00	0.00	0.00001	0.00001
a8	0.00009	0.000044	0.00	0.00	0.00001	0.00018
a9	−0.00000	0.000000	0.00	0.00	−0.00000	−0.00000

**Table A15:** The statistical parameters of polynomial regression analysis of the influence of SS torrefaction temperature and residence time on the Mg content in biochars.

Mg, mg·kg^−1^						
Model	Mg, mg·kg^−1^ = (1051.47) + (0.247677)·T^2^ + (−47.1368)·T + (5.53494)·t^2^ + (−538.67)·t + (10.2288)·T·t + (−0.0310345)·T^2^·t + (−0.122493)·T·t^2^ + (0.000382962)·T^2^·t^2^ R^2^ = 0.50					
Function Parameters	Value	Standard Error	t Value	*p* Value	Lower Confidence Limit	Upper Confidence Limit
a1	94.32113	57.65115	0.00	0.00	−20.3866	209.0288
a2	0.00132	0.00093	0.00	0.00	−0.0005	0.0032
a3	−0.64647	0.46742	0.00	0.00	−1.5765	0.2835
a4	0.11022	0.04050	0.00	0.00	0.0296	0.1908
a5	−7.32599	3.27329	0.00	0.00	−13.8388	−0.8132
a6	0.06125	0.02654	0.00	0.00	0.0084	0.1141
a7	−0.00013	0.00005	0.00	0.00	−0.0002	−0.0000
a8	−0.00091	0.00033	0.00	0.00	−0.0016	−0.0003
a9	0.00000	0.00000	0.00	0.00	0.0000	0.0000

**Table A16:** The statistical parameters of polynomial regression analysis of the influence of SS torrefaction temperature and residence time on the Ca content in biochars.

Ca, mg·kg^−1^						
Model	Ca, mg·kg^−1^ = (316658) + (5.82666)·T^2^ + (−2648.24)·T + (341.228)·t^2^ + (−23391.2)·t + (213.523)·T·t + (−0.468153)·T^2^·t + (−3.09572)·T·t^2^ + (0.00673435)·T^2^·t^2^ R^2^ = 0.32					
Function Parameters	Value	Standard Error	t Value	*p* Value	Lower Confidence Limit	Upper Confidence Limit
a1	316658.0	485972.1	0.65	0.52	−680474	1313790
a2	5.8	7.9	0.74	0.47	−10	22
a3	−2648.2	3936.8	−0.67	0.51	−10726	5429
a4	341.2	336.5	1.01	0.32	−349	1032
a5	−23391.2	27228.4	−0.86	0.40	−79259	32477
a6	213.5	220.5	0.97	0.34	−239	666
a7	−0.5	0.4	−1.06	0.30	−1	0
a8	−3.1	2.7	−1.14	0.27	−9	2
a9	0.0	0.0	1.24	0.23	0	0

**Table A17:** The statistical parameters of polynomial regression analysis of the influence of SS torrefaction temperature and residence time on the K content in biochars.

K, mg·kg^−1^						
Model	K, mg·kg^−1^ = (−7811.08) + (0.0132002)·T^2^ + (35.0213)·T + (−5.9302)·t^2^ + (432.779)·t + (−0.0448789)·T·t + (−0.00580631)·T^2^·t + (0.002207)·T·t^2^ + (7.25633 × 10^−5^)·T^2^·t^2^ R^2^ = 0.48					
Function Parameters	Value	Standard Error	t Value	*p* Value	Lower Confidence Limit	Upper Confidence Limit
a1	−7811.08	33686.23	−0.23	0.82	−76929.5	61307.36
a2	0.01	0.55	0.02	0.98	−1.1	1.13
a3	35.02	272.89	0.13	0.90	−524.9	594.95
a4	−5.93	23.33	−0.25	0.80	−53.8	41.93
a5	432.78	1887.38	0.23	0.82	−3439.8	4305.37
a6	−0.04	15.28	0.00	1.00	−31.4	31.32
a7	−0.01	0.03	−0.19	0.85	−0.1	0.06
a8	0.00	0.19	0.01	0.99	−0.4	0.39
a9	0.00	0.00	0.19	0.85	0.0	0.00

**Table A18:** The statistical parameters of polynomial regression analysis of the influence of SS torrefaction temperature and residence time on the Na content in biochars.

Na, mg·kg^−1^						
Model	Na, mg·kg^−1^ = (−41466.8) + (−0.577248)·T^2^ + (318.433)·T + (−49.4931)·t^2^ + (3620.2)·t + (−27.0631)·T·t + (0.0542841)·T^2^·t + (0.390176)·T·t^2^ + (−0.000809744)·T^2^·t^2^ R^2^ = 0.29					
Function Parameters	Value	Standard Error	t Value	*p* Value	Lower Confidence Limit	Upper Confidence Limit
a1	−41466.8	141846.8	−0.29	0.77	−332512	249,578.7
a2	−0.6	2.3	−0.25	0.80	−5	4.1
a3	318.4	1149.1	0.28	0.78	−2039	2676.2
a4	−49.5	98.2	−0.50	0.62	−251	152.0
a5	3620.2	7947.5	0.46	0.65	−12,687	19,927.1
a6	−27.1	64.4	−0.42	0.68	−159	105.0
a7	0.1	0.1	0.42	0.68	0	0.3
a8	0.4	0.8	0.49	0.63	−1	2.0
a9	0.0	0.0	−0.51	0.61	0	0.0
